# Cross-Sectional Risk Factors of Anterior Knee Pain in Adolescents

**DOI:** 10.3389/fpain.2021.720236

**Published:** 2021-10-22

**Authors:** Gregory Borschneck, Laura St. John, Kristy Brundage, Daniel Patrick Borschneck

**Affiliations:** ^1^Department of Biomedical and Molecular Sciences, Faculty of Health Sciences, School of Medicine, Queen's University, Kingston, ON, Canada; ^2^Infant and Child Health Lab, Faculty of Kinesiology and Physical Education, University of Toronto, Toronto, ON, Canada; ^3^Faculty of Health Sciences, School of Rehabilitation Therapy, Queen's University, Kingston, ON, Canada; ^4^Department of Surgery, Faculty of Health Sciences, School of Medicine, Queen's University, Kingston, ON, Canada

**Keywords:** patella-femoral pain syndrome, anterior knee pain (AKP), sport, pediatric pain, joint pain, chronic pain, musculoskeletal pain

## Abstract

**Background:** Anterior knee pain is a common complaint amongst adolescents, which can both be persistent, and in some cases, disabling. This study investigated a series of potential risk factors potentially linked to the onset of anterior knee pain.

**Methods:** Questionnaires were distributed amongst 367 10–15 years-olds enrolled in the local school board. These surveys included questions on sex, age, sport participation, and history of anterior knee pain verified by a physician. Bivariate correlations and a binomial logistic regression were conducted. Overall rate of AKP in the population studied was 7.4%. The results indicated that past history of knee pain, age, and increased sports participation significantly correlated with increased risk of AKP. AKP was significantly more common in females than males. While sex, height, age, overall sport participation, participation in specific sports, and history of knee injury all contributed to the binomial model.

## Introduction

Anterior knee pain (AKP) describes a group of persistent (1) and occasionally disabling conditions afflicting the anterior aspect of either one or both knees (2). AKP is often used interchangeably with the patellofemoral joint pain (PFJP) (3), but also encompasses broader conditions including patellar tendinopathy, Osgood Schlatter's disease, and pain of the anterior tibiofemoral joint. AKP is relatively uncommon in children (<10 years old) (4, 5); however, prevalence significantly increases throughout adolescence impacting 20–30% of this population (5, 6). Adolescents often report this pain as mild; however, in some cases (13%) it may be a significant disability (5) associated with early-onset patellofemoral osteoarthritis (7). AKP is responsible for between 10 and 40% of all physiotherapy visits made by adolescents (8, 9) and often results in maladaptive behaviors including withdrawal from sports (10). Despite this prevalence and severity, the majority of research on AKP takes place in adults rather than adolescents (11).

AKP has been linked with poor biomechanical alignment across the trunk, hip, knee, and ankle joints (12). Numerous intrinsic factors are thought to relate with poor alignment, including lack of flexibility (13), poor muscular strength (14, 15), natural alignment (15, 16), and poor motor control (12, 17); such patterns and high-risk malalignment is known to be particularly common amongst females (12). While intrinsic risk factors surrounding alignment have received considerable focus in recent meta-analyses (18, 19), it is also important to consider how extrinsic risk factors related to loading quantity relate to AKP rates. Amongst adolescents, organized sporting participation is known to increase (20); those involved in sports throughout adolescence demonstrate increased levels of overall, moderate, and vigorous physical activity in both sexes (less prominent in girls) (21). Despite the link between overall loading and AKP risk, past cross-sectional studies have not demonstrated a consistent link between sports participation status and AKP development in adolescents (5, 6, 22). These studies did not consider type of sport, only overall sporting behavior; as some sports have a known association with AKP (23, 24) it is possible that cultural differences in prevalence of specific sports has impacted this relationship.

Accordingly, the purpose of this study was to determine if overall sporting participation or involvement in specific sports place adolescents at an increased risk of AKP.

## Methods

### Population

**Figure 1:**
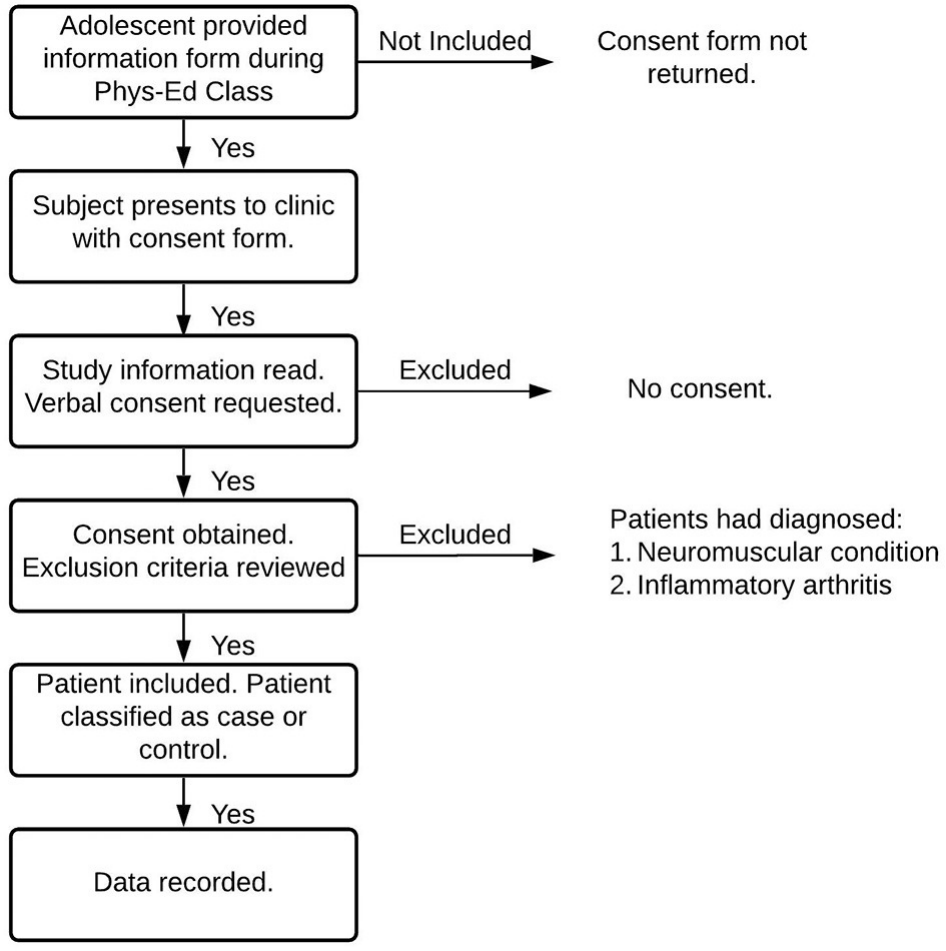
Inclusion Criteria Flowchart. During their Phys. Ed. class, adolescents were provided an information form regarding the protocols and purpose of the current study. Written, informed parental consent and adolescent assent were acquired at a subsequent clinic. Adolescents with diagnosed inflammatory arthritis or any diagnosed neuromuscular conditions were excluded from the study.

A cross-sectional survey was performed between May 2001 to June 2001. Participants were a convenience sample of pre-adolescent and adolescent youth (10–15 years) enrolled in three public primary schools and two public secondary schools throughout Kingston, Ontario, Canada. Following the permission of the schools, students in randomly selected physical education classes were approached in the presence of their teachers; these students were handed a brochure explaining the study, a parental consent form, and data questionnaire (Figure 1). The population of students sampled excluded those who were not enrolled in general classes due to special needs requirements, were diagnosed with neuromuscular conditions, or inflammatory arthritis; study designers were blind to any prevalence of AKP during selection. Prior to data acquisition, ethics approval was obtained from both the Queens University and Affiliated Teaching Hospitals Health Sciences Research Ethics Board and the Limestone District School Board. Participants and parents were informed about the purpose and methodology of the study; informed written consent was obtained from the parent and informed verbal assent from the child before enrollment. Only students who verbally assented to inclusion and returned both their completed surveys and parent consent forms were considered for participation in this study.

### Metrics

Demographic information including age, height, and sex were recorded. History of medically diagnosed knee injury and the dichotomous presence/absence of knee pain localized to the anterior knee which was significant enough to limit activity for more than one week over the past year were also documented. Participants were asked via an open-ended text box to list the sporting activities (outside of physical education class) that they spent a significant amount of time participating in over the past year; it was hoped that allowing a participant to subjectively determine the number of sports that they participated in would allow them to include any unorganized or “pick-up” sports they played. An additional combinate categorical variable termed “Total Number of Sports” (reflecting the total number of sports the adolescent played) was also analyzed; due to the limited number of athletes playing four (11), five (7), or six (3) sports, these subjects have been grouped into a single category as ≥3.

### Procedure

Students completed a self-report questionnaire, which was returned to the research team. Upon return of the self-report questionnaire, a physician (who was a research team member) was available to aid in question comprehension, ensure accurate responses, and gather missing data. Presence of AKP, based on the definition listed in section 2.2 was confirmed during the follow up interview. Height was recorded for each subject following return of survey and consent form.

### Statistical Analysis

All statistical analyses were performed using SPSS 27.0.1.0. Any subjects with missing data were excluded from the analysis. Dichotomous independent variables sex, participation in any sports and binary participation in specific sports were analyzed along with the binary dependent variable of knee pain in an exact Fisher's Test. An exact Fisher's test was chosen due to concerns that some groups would involve fewer than five participants. Along with the significance of the exact Fisher's test, the phi coefficient was recorded as a measure of association. The ordinal variable total sport involvement was analyzed against knee pain using a Pearson's Chi-Square analysis due to the non-binary nature of this data. Continuous independent variables height and age were tested for association against the independent variable of knee pain using a Spearman's rho correlation; significance and correlation coefficient.

A binomial logistic regression was conducted due to the binary nature of the dependent variable. All variables recorded in the study were initially included and remained in the model if they contributed according to the results of the wald test. The binomial logistic regression method was chosen because multiple independent variables were considered, and due to the dichotomous nature of the dependent variables measured. A correlation between predictor variables was run alongside the regression to confirm that the assumption of independence was upheld; in this correlation the combined metric “total sport involvement” was found to moderately correlate with several other factors and so was removed from the hierarchical regression.

## Results

The final study sample included 367 participants (see Table 1).

**Table 1:** Demographics.

**Descriptor**	***N***
Total Subjects	367
Sex	
Females	207
Males	160
Age group (years)	
10	11
11	44
12	52
13	57
14	149
15	55
Current number of extra-curricular sports played	
0	280
1	28
2	38
3	17
≥3	21
Anterior Knee Pain	
Yes	27
No	340
Knee injury	
Yes	18
No	349

*Overall, 367 participants were included in this study, There were more females (n = 207) than males (n = 160) in this study's sample. The greatest number of participants were 14 years old (n = 149), while the fewest were 10 years old (n = 11). The majority of participants in this study did not play sports (n = 280). AKP was reported in 27 participants*.

### Demographics

There were significantly more females (*n* = 207) than males (*n* = 160) enrolled; the mean age of the sample was 13.2 years (SD = 1.36), with a range from 10 to 15 years. Twenty-seven of the participants (7.4%) reported knee pain, and 18/367 (4.9%) reported a knee injury in the previous year. Of the 367 participants, 87 (23.7%) of the participants were involved in extracurricular sports with the most commonly played sports being basketball (*n* = 31), soccer (*n* = 29), and swimming (*n* = 27). Descriptive characteristics are further explained on Table 1.

Incidence of AKP was greatest amongst 14-year-olds (10.74%) and non-existent amongst 10-year-olds (0.00%). AKP was most prevalent amongst those with a history of knee injury (61.11%) and generally increased in those who played any sport (22.99%). Table 2 displays specific incidence for each group.

**Table 2:** Incidence per Group.

**Variable**	**Total count**	**Percent with AKP**
Age^†^	367	7.36%
10	11	0.00%
11	44	2.27%
12	52	5.77%
13	57	3.51%
14	149	10.74%
15	55	9.09%
Sex^†^	367	7.36%
Male	160	3.75%
Female	207	10.14%
Knee Injury	18	61.11%
Total Sport^††^	87	22.99%
0	280	2.50%
1	28	21.43%
2	38	15.79%
3	17	23.81%
>3	21	38.10%
Sports^††^	87	22.99%
Soccer	29	17.24%
Basketball	31	29.03%
Baseball	17	29.41%
Hockey	14	7.14%
Swimming	27	11.11%
Cross-Country	14	28.57%
Riding	4	50.00%
Skating	7	14.29%
Volleyball	25	28.00%

*This table depicts incidence of AKP across the different groups in this study along with the total count for the given condition. Highest prevalence was amongst those with past knee injuries. Incidence of knee pain notably increases with age and amongst females*.

*AKP, Anterior Knee Pain*.

† *Rate of AKP reported for entire sample*;

†† *Rate of AKP reported for all engaged in sport*.

### Individual Associations

Statistically significant associations were observed between both AKP and sex (*r* = −0.121, *p* = 0.02), AKP and age (*r* = 0.112, *p* = 0.033), and AKP and total number of sports played (*r* = 0.329, *p* < 0.001). Females (*r* = 0.121, *p* = 0.02) were more likely to experience knee pain than males. A moderate risk for knee pain was noted among those involved in extracurricular sports (*r* = 0.352, *p* < 0.001) compared to those not participating in sports. A moderately increased risk for knee pain was observed in participants with a history of knee injury (*r* = 0.468, *p* < 0.001). Height was not found to significantly relate to risk of developing AKP (*p* = 0.646) both overall and after controlling for participant age.

Participation in soccer (*r* = 0.111, *p* < 0.034), basketball (*r* = 0.252, *p* < 0.001), baseball (*r* = 0.186, *p* = 0.005), cross-country (*r* = 0.162, *p* = 0.014), riding (*r* = 0.171, *p* = 0.029), and volleyball (*r* = 0.214, *p* = 0.001) all were found to slightly but significantly increase risk of developing AKP. Total sports played (*r* = 0.352, *p* < 0.001) significantly positively correlated with increased rates of AKP. When sporting involvement was controlled for, participation in soccer, football, hockey, swimming, gymnastics, and skating all correlated slightly negatively with AKP, whereas participation in basketball, baseball, cross-country, riding, and volleyball correlated slightly positively with AKP.

### Binomial Logistic Regression

A binomial logistic regression was performed to predict knee pain based on age, sex, history of knee injury, height, number of sporting activities, and the dichotomous nature of all sports recorded. As the only continuous variables involved in this regression, height and age were tested a priori using the Box-Tidwell transformation to confirm that there was no violation of the assumption of the linearity of logit. Football and gymnastics were subsequently removed from this model as they returned Wald statistics of 0. This binomial logistic regression was a statistically significant improvement (*p* = 0.00) over an unconditional model.

When all variables in this study were accounted for, history of knee injury (OR = 18.422, *B* = 2.914) and total sporting involvement at all levels (one sport: OR = 19.019, *B* = 2.945; two sports: OR = 75.585, *B* = 4.814; three sports: OR = 165.756, *B* = 6.134; 4 sports: OR = 9,684.151; *B* = 9.665) remained significant (Table 3). Two sports were significant in the model, participation in swimming (OR = 0.047, *B* = −3.060) and soccer (OR = 0.018, B = −4.005) were both found to demonstrate a protective effect on AKP. All metrics involved in the model are described in Table 3.

**Table 3:** Binary logistic regression results.

**Variable**	**B**	**SE**	***P***	**OR**	**95% CI**	
					**Lower**	**Upper**
Constant	−2.533	0.200	<0.001^**^	0.79	–	–
Age	0.372	0.284	0.190	1.451	0.832	2.531
Sex^†^	0.033	0.676	0.961	1.034	0.275	3.889
Height	−0.020	0.032	0.533	0.980	0.921	1.044
Total Sport Participation		0.003^*^				
1 Sport	2.945	0.816	<0.001^**^	19.019	3.839	94.216
2 Sports	4.814	1.347	0.001^*^	75.585	5.393	1059.375
≥3 Sports	6.134	2.059	0.008^*^	165.756	2.927	9386.299
4 Sports	9.665	3.213	0.006^*^	9,684.151	17.823	5261828.97
Knee Injury	2.914	0.739	<0.001^**^	18.422	4.327	78.438
**Individual Sport Participation**						
Basketball	−0.800	1.168	0.493	0.449	0.046	4.429
Baseball	−0.857	0.985	0.384	0.424	0.062	2.925
Cross-Country	0.388	1.224	0.751	1.475	0.134	16.226
Hockey	−2.324	1.406	0.098	0.098	0.006	1.541
Riding	−0.625	1.769	0.724	0.535	0.017	17.171
Skating	−1.751	2.322	0.451	0.174	0.002	16.433
Soccer	−4.005	1.899	0.035^*^	0.018	0.000	0.753
Swimming	−3.060	1.243	0.014^*^	0.047	0.004	0.536
Volleyball	−1.448	1.227	0.238	0.235	0.021	2.605

*Total sporting activity and history of knee injury were significantly related to presence and increased risk of AKP. Participation in one of hockey, swimming, and soccer was found to serve a significantly protective effect against AKP. Football and dance demonstrated a Wald statistic of zero and accordingly were not included in this table*.

*OR, Odds Ratio; CI, Confidence Interval*.

† *Sex data is relative to Males*.

* *p < 0.05*;

** *p < 0.001*.

## Discussion

The current study sought to identify risk factors associated with and rates of AKP in pre-adolescents and adolescents, representing one of the largest cross-sectional samples to date. AKP's prevalence in this study (7.4%) was lower than other reports on the general adolescent population. A study by Fairbank described self-reported knee pain at a prevalence of 30% within a population aged 13–17 years old; this study focused on patellofemoral joint pain but also included other conditions such as Osgood-Schlatter's disease (6). Vahasarja reported the prevalence of a generalized anterior knee pain in 18.5% of their sample aged 14–15 years (5). AKP was most prevalent in our study's 14-year-old population, of which 10% had AKP; this prevalence is lower than reported by Fairbanks and Vahasarja. As both the Fairbanks and Vahasarja studies were conducted in Europe more than 5 years before this study, perceptions surrounding pain may differ. It is unclear whether these temporal variations and subtle differences in descriptions and categorization of pain impacted reported prevalence.

### Age

This study found a slight but significant positive relationship between age and prevalence of AKP. Prevalence of AKP within the sample was much greater amongst participants 14 and older (10.37%) compared to those age 13 and younger (3.89%). Vähäsarja (5) generally agree with such a finding, demonstrating that postpubescent (14–15-year-olds) present with a significantly increased rate of knee pain compared to prepubescent children (9–10 years old). There are many potential explanations for this finding ranging from the onset of puberty to the accumulation of damage.

### Sex

Females' tendency to report an increased prevalence of knee pain is consistent with past observations (25–27). Increased prevalence of AKP in female adolescents not only exists in the general population but also among athletes. Amongst pubertal females, tissue properties are known to associate with biomechanical changes; these changes may associate with the increased incidence of AKP this study found in adolescent females (28).

Alike in the bivariate analysis being a female increased risk of reporting AKP in the linear model; however, this effect was not significant. This may be due to a statistically significant increase in the prevalence of past knee injuries amongst female participants and a statistically significant decrease in hockey involvement, which was one of the sports that demonstrated protective effects against AKP development.

### Sport Participation

Participation in organized sports was moderately associated with knee pain. This finding is not consistent with the findings of Fairbank, who did not identify a sports/knee pain association (6). Fairbank inquired about sports involvement by asking respondents “Do you like to play sport” with the possible responses: “as much as possible,” “average,” “as little as possible.” Forty-five percent of respondents were in the first category “as much as possible,” and 50% played an average amount (6). The structure of the Fairbank study question may have led to misclassification of this sports involvement variable. Many adolescents may wish to play sports “as much as possible” but play little in reality, making comparisons difficult. Generally, studies that focus on elite athletic pediatric populations demonstrate an increased study-wide incidence of AKP compared to these broader studies.

While increasing sport participation was found to increase risk of AKP (increasing OR, see Table 3) a matched-cross-sectional study by Rathleff et al. disagrees, finding that those with AKP withdrew, reducing their sporting participation (29). As the sample Rathleff et al. tested was considerably older (ages 15–19) than those assessed in this study this disagreement may reflect a temporal relationship between sport and AKP. Specifically, the marked increase in AKP incidence once subjects reach 14 years of age (Table 2) may lead to the increased dropout that Rathleff et al. reported between years 15–19 (29).

This study is one of few to examine which sports may increase or decrease risk of AKP within the same population. Research regarding sport-based prevalence of AKP have focused primarily on a select few sports, such as dance. The prevalence of AKP across both the general adolescent population and all sports studied here urges authors to consider how under-researched activities like horseback riding relate to AKP risk and compare to other sports.

### Knee Injury

Knee injury within the past year was found to significantly associate with reported AKP both in bivariate assessments and linear modeling. A major systematic review of all research in this field suggested that several of the biomechanical patterns known to increase risk of AKP are particularly common in injured knees (30). As outlined above rate of knee injury within the past year was found to be significantly greater amongst females. These results concur with past research which suggests that in most sports females are equally or more likely to experience knee injuries -particularly ACL tears- than males (31).

### Limitations

One limitation of this study was its population. As a true cross-sectional study the number of participants with AKP was considerably lower than those unaffected, limiting the generalizability of these findings. The disproportionate nature of the control and AKP groups were to be expected in the population of interest. While this study examined the overall number of sports a participant played, it did not account for the hours and rigor involved in that activity. Accounting for sporting intensity may help explain why risk of AKP changes relatively little after an athlete is already engaged in sport; this would better account for the risk of AKP faced during early specialization (32–34). It is important to note that a small percentage (31%) of our population was engaging in extracurricular sport which greatly differs from what has been seen in Canadian Youth (35). While this disparity could impact the generalizability of findings, it does reflect the population that we sampled who tended to be in their young teens when sport drop out occurs more frequently (36, 37). Lastly, the data involved in this study was collected in 2001 and any cultural changes surrounding pain or sporting behavior since then may impact generalizability.

Despite these limitations, the results of this study are largely generalizable. The disproportionate number of females in the study population was controlled for during modeling. Further, as all adolescents are required to take physical education at the ages sampled there is no reason to suggest that this study was not open to the general population of adolescents in the grades sampled.

## Conclusions

Analysis revealed that the most potent risk factor for AKP pain is past, treated, knee injuries which seemed to predispose females in particular to the development AKP. Athletes who participated in some sports were at heightened risk of developing AKP, although this did not scale with the number of sports played. While non-significant, different sports variably influenced risk of AKP development; future studies should look to compare these sports with a larger sample size to determine how involvement impacts overall risk. Future studies should attempt to assess how pain severity relates to the factors evaluated in this study; understanding how different groups of adolescents respond to different severities of AKP may help develop early warning signs for this condition.

## Data Availability Statement

The original contributions presented in the study are included in the article/Supplementary Material, further inquiries can be directed to the corresponding author/s.

## Ethics Statement

The studies involving human participants were reviewed and approved by Queens University and Affiliated Teaching Hospitals Health Sciences Research Ethics Board. Written informed consent to participate in this study was provided by the participants' legal guardian/next of kin.

## Author Contributions

GB performed statistical analysis based on data obtained within DB's thesis. GB and LS wrote and edited the manuscript with consult from DB. KB was integral to data collection. DB conducted the original thesis from which this study is derived. All authors contributed to the article and approved the submitted version.

## Conflict of Interest

The authors declare that the research was conducted in the absence of any commercial or financial relationships that could be construed as a potential conflict of interest.

## Publisher's Note

All claims expressed in this article are solely those of the authors and do not necessarily represent those of their affiliated organizations, or those of the publisher, the editors and the reviewers. Any product that may be evaluated in this article, or claim that may be made by its manufacturer, is not guaranteed or endorsed by the publisher.
